# Natural Frequency Transmissibility for Detection of Cracks in Horizontal Axis Wind Turbine Blades

**DOI:** 10.3390/s24144456

**Published:** 2024-07-10

**Authors:** Rachel Henderson, Fae Azhari, Anthony Sinclair

**Affiliations:** Department of Mechanical and Industrial Engineering, University of Toronto, 5 King’s College Road, Toronto, ON M5S 3G8, Canada; rachelc.henderson@mail.utoronto.ca (R.H.); azhari@mie.utoronto.ca (F.A.)

**Keywords:** horizontal axis wind turbine, vibration, transmissibility, natural frequency, non-destructive examination, condition monitoring, acceleration, strain, 3D printing

## Abstract

Defects on horizontal axis wind turbine blades are difficult to identify and monitor with conventional forms of non-destructive examination due to the blade’s large size and limited accessibility during continuous operation. This article examines both strain and acceleration transmissibility as methods of continuous damage detection on wind turbine blades. A scaled 117 cm offshore wind turbine blade was first designed, 3D printed, and modelled numerically in ANSYS. Transverse cracks were deliberately introduced to the blade at 10 cm intervals along its leading edge. Subsequent changes in the transmissibility, relative to an undamaged baseline model, were measured using different variable combinations at the blade’s first three natural frequencies. Experimental results indicated that strain transmissibility was able to locate a 1.0 cm defect at a range of 70–110 cm from the blade hub using the amplitudes of the first natural frequency of vibration. The numerical model was able to simulate the strain experimental results and was determined to be valid for future defect characterization. Acceleration transmissibility was unable to experimentally identify defects sized at 1.0 cm and below but was able to identify 1.0 cm sized defects numerically. It was concluded that transmissibility is viable for continuous damage detection on blades but that further research into other defect types and locations is required prior to conducting full-scale testing.

## 1. Introduction

### 1.1. Objectives

This research focuses on the use of natural frequency transmissibility as an online method of damage detection on horizontal axis wind turbine blades. Given a wind turbine blade’s large size and limited accessibility while in service, challenges exist with online monitoring and defect characterization while damage is still in its infancy. It is important for the wind turbine blades to be continuously monitored as many damage mechanisms for wind turbines are random in nature and are not time-based. Extensive repairs, replacements, and wind turbine failure can be mitigated with an appropriate structural health monitoring program combined with timely maintenance.

Our investigation centred on changes in the vibration transmissibility between pairs of sensors due to a transverse defect added to the leading edge of the blade. Single defects were induced at different locations along the blade’s length and then assessed using both strain and acceleration transmissibility. The transmissibility changes between pairs of sensors were assessed as functions of several different variables, including modal frequency, signal type, and sensor orientation. The intent was to determine parameter settings that maximized the damage detection capabilities of transmissibility. This was completed by comparing the results of different variable combinations using a confusion matrix analysis. The objective of this research was to determine an approximate range of defect size and location on the blade for which the defect could be recognized with the best performing variable combination. An additional objective of this research was the development and validation of a numerical model to allow for additional types of damage to be characterized by transmissibility methods in future work.

This project expands on past transmissibility research by focusing on the transmissibility changes at each natural frequency individually as opposed to utilizing the entire frequency spectrum or a summation of the natural frequencies. It develops a damage identification algorithm to decide if a defect has occurred and determine the location. It provides an analysis of the effect of the defect location on the ability to identify the damage with transmissibility. It also examines the effects of the sensor orientation as well as the signal type used for analysis. Furthermore, this project utilizes smaller, more realistic-sized defects that would not be immediately detrimental to blade integrity and would be difficult to identify with conventional structural health monitoring methods.

### 1.2. Background and Literature Review

#### 1.2.1. Wind Turbine Blades

**Figure 1:**
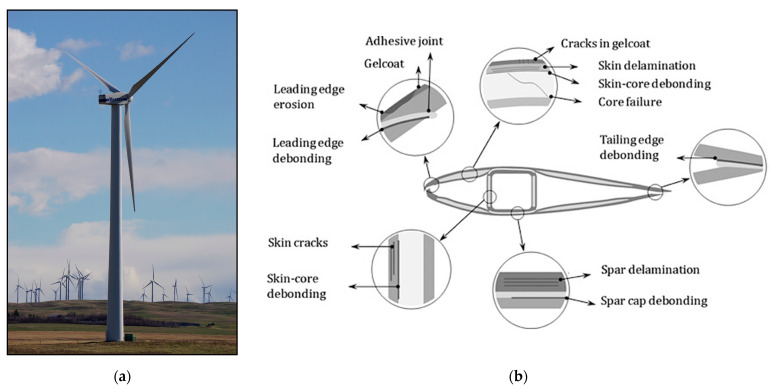
(**a**) Horizontal axis wind turbine at a wind farm near Pincher Creek, Alberta, Canada. (**b**) Typical defects shown on a wind turbine blade’s cross section. Reproduced with permission from [19]. Katnam et al., Journal of Adhesion; published by Taylor & Francis Ltd., 2014.

Horizontal axis wind turbines (HAWT) have become a symbol of renewable energy generation in Canada and across the world. In 2022, approximately 6.6% of the electricity demand in Canada was supplied by wind power [1]. Many projects are currently in development to increase the amount of energy produced by wind in Canada. This includes onshore projects that are currently under construction such as the 200 MW Buffalo Atlee project in Alberta [2] and the 200 MW Bekevar Wind Facility in Saskatchewan [3]. The provinces of Nova Scotia and Newfoundland have recently made steps toward the development of offshore wind in Canada by amending existing legislation to regulate offshore renewable energy projects [4]. Nova Scotia has also announced plans to provide leases for 5 GW of offshore wind energy by 2030 [5]. Wind turbines are, therefore, an important part of Canada’s current and future energy outlook. A typical large-scale horizontal axis wind turbine is shown in Figure 1a.

Many wind turbines used in large-scale energy production, such as Vestas [6,7], Siemens Gamesa [8], GE [9], and Enercon [10], are designed for a 20–30 year lifespan before decommissioning, replacement in kind or repowering is required. In Canada, approximately one-third of the wind turbines listed in the Canadian Wind Turbine Database are set to reach an age of 20 or older by 2030 [11]. As turbines approach their end of life, the components are more likely to experience damage and degradation. Inspection and timely maintenance of wind turbine components are therefore important to identify and mitigate damage to keep the turbine online and produce electricity.

Wind turbine blades are prone to damage, which can affect the overall availability and efficiency of the turbine over time. The author in [12] referenced blade data from the insurer GCube, stating that blades had an annual failure rate of 0.54%, with over 40% of wind turbine insurance claims occurring as a result of blade failure. Replacement blade costs are estimated at USD 80,000–USD 300,000 as of 2023 [13]. Blade repair costs are significantly cheaper at estimates of USD 20,000 for a typical repair. Repair cost is, however, dependent on the severity of the damage.

Several reviews of wind turbine blade damage types and causes were conducted in [14,15,16,17]. Common damage seen in blades includes leading edge erosion, leading and trailing edge bond splits, spar cracking/debonding, fatigue cracking, and delaminations. Damage can occur resulting from regular operation, with leading edge erosion and fatigue cracking probability becoming more prevalent the longer the blade is in service. Operating incidents, including high wind events, hail, icing, lightning, debris and bird strikes, may also contribute to blade damage. Some defects, such as delaminations, voids, fibre misalignment, lack of fusion, kissing bonds, and wrinkles, can occur in the initial manufacturing process [18]. These imperfections can propagate and become problematic once in service. Defects can be both external or internal to the blade, as shown in Figure 1b [19].

#### 1.2.2. Inspection Methods

Vestas reports that an inspection costs 0.001 percent of a blade replacement and can reduce repair costs by upwards of 75% if the damage is caught early [20]. The majority of inspections that occur on wind turbine blades in-service are visual. Visual inspections can be completed with technicians via crane or rope access. Inspections can also be remotely completed with high powered cameras and drones. Industrial companies have been able to recognize defects as small as a few millimetres with remote visual methods [21,22]. Artificial intelligence has also been used in research to identify defects from remote visual inspection using image recognition [23]. Although effective for the detection of surface damage both externally and internally, visual inspections can be time consuming and cannot detect subsurface damage within the blade’s structure.

Passive thermography can examine the external and internal components of the blade via camera or drone and can be effective at identifying many different types of defects, especially when combined with visual inspection. The research group in [24,25] examined a blade segment exposed to different ambient conditions. Their work was successful at recognizing different types of defects using thermograms, but they noted the dependence on time of day, temperature and weather conditions. Different types of defects were individually more or less apparent on the thermogram, depending on these parameters at the time of the inspection. Multiple inspections would, therefore, be required throughout the day in ideal weather conditions to properly examine the blade for all damage types.

Ultrasonic testing (UT) is another method that can be used for defect detection on wind turbine blades. This includes conventional pulse-echo and phased array [26], guided wave [27] and automated methods [28]. Although UT is well-established and can identify many types of defects, ultrasound is impractical in service, given the amount of time required to perform a complete blade scan. Tests of the conventional pulse-echo in [26] found an average scan rate of 11:48 $min/{ft}^{2}$ on composite panels representing different parts of a blade. Phased array and automated methods would be much faster to use but would still take significant time, given the size of most wind turbine blades. Guided wave UT was effective at detecting disbonds on wind turbine blades, as shown in [27], and is ideal for longer distances. Guided wave UT is still primarily in the research phase for wind turbine blades. In general, UT is more prevalent in industry for the identification of factory defects with ultrasound before the blade is put into service.

The above non-destructive examination methods, although effective, only represent the blade’s condition at a moment in time. As a wind turbine is in constant operation and many of its damage mechanisms are not time dependent, a continuous inspection method is preferable. Continuous methods of blade monitoring include acoustic emission, auditory monitoring with microphones and vibration. SCADA data analysis can also be used to scan abnormalities in the blade using operating data, for example, blades exposed to lightning strikes [29].

Acoustic emission (AE) has been the subject of numerous research studies, such as [30,31]. There are also commercial products available that use AE on blades, such as Sensoria, offered by MISTRAS [32]. Although effective as a continuous monitoring method at identifying propagating defects as well as defect location, AE struggles with assessing defect characterization and severity. It is also prone to noise from the environment and requires complicated signal processing methods to assess the signal [33].

Research in [34] examined passive acoustic monitoring in wind turbine blades. This method utilized microphones measuring changes in the acoustic transmissions within the structure when exposed to different through-wall defects. This method was able to identify different severities but noted different results for a defect based on its location on the airfoil’s cross section. This research is promising for a structural health monitoring method as it is low-cost and easy to implement. This method would be reliant on defects being through-wall, so damage severity may be more severe at the time of discovery versus other methods.

Vibration condition monitoring systems are available in the industry for blades, such as Weidmüller’s BLADEcontrol system [35]. Most of the research on vibration monitoring of wind turbine blades is passive-based and relies on excitation occurring due to the wind. In [36,37], operational modal analysis techniques were used for damage detection in blade simulations. Active vibration monitoring using an actuator was also conducted on an operating Vestas turbine in [38] and was able to recognize a trailing edge crack.

Vibration condition monitoring programs often focus on monitoring the amplitudes of a raw signal or changes to the natural frequencies of the component. Identification of large changes in the amplitude of a raw vibration signal can provide protection from operating events as the turbine can be shutdown to minimize significant damage. Large structures, such as wind turbine blades, tend to have minimal natural frequency changes at the lower modes when exposed to damage. Damage detection is, therefore, difficult unless the damage is severe. Furthermore, locating the defect can be a challenge with more common vibration methods.

The inspection methods discussed above have their limitations. Visual inspection, thermography and ultrasound are primarily discontinuous and periodic inspections may fail to capture blade damage until it has propagated to a major repair or failure. Continuous methods such as SCADA analysis, acoustic emission, and vibration are effective but require significant damage to alert the operator of an issue. Characterization of the defect is also difficult with continuous methods. AE is the only method of the three that can determine location but is prone to environmental noise. A subset of vibration, transmissibility, aims to provide better sensitivity to damage than traditional vibration analysis using amplitudes and natural frequencies. Transmissibility is a continuous method of NDE, making it highly advantageous for equipment in operation. It aims to identify and localize the defect and provide that information to the wind turbine operator to assess the risk to the blade’s integrity.

#### 1.2.3. Transmissibility Theory of Single and Multiple Force Functions

The transmissibility theory of multi-degree-of-freedom (MDOF) systems, as well as the application to damage detection, is discussed extensively in [39,40,41,42,43]. Vibration transmissibility describes the relationship between an output response and an input function. In an MDOF system, transmissibility is calculated by determining the ratio of the output responses between two locations on the same structure. Research has shown that the transmissibility between a pair of sensors can be independent of the applied force input; this suggests its potential as a tool for non-destructive evaluation (NDE) of in-service engineering structures.

**Figure 2:**
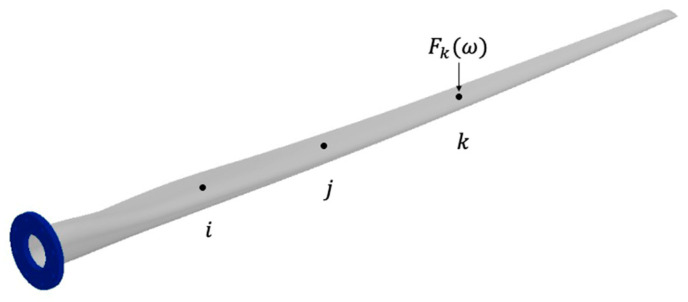
Wind turbine blade with a force applied at location k.

Consider the wind turbine blade in Figure 2. In this figure, $X_{i}\left(\omega \right)$ and $X_{j}\left(\omega \right)$ are the measured output responses at positions i and j resulting from an applied forced $F_{k}\left(\omega \right)$ at position k.

(1) $$X_{i}\left(\omega \right)=H_{ik}\left(\omega \right)F_{k}\left(\omega \right)$$

(2) $$X_{j}\left(\omega \right)=H_{jk}\left(\omega \right)F_{k}\left(\omega \right)$$

(3) $$T_{ijk}(\omega )=\frac{X_{i}(\omega )}{X_{j}(\omega )}=\frac{H_{ik}(\omega )F_{k}(\omega )}{H_{jk}(\omega )F_{k}(\omega )}=\frac{H_{ik}(\omega )}{H_{jk}(\omega )}$$

The transmissibility, $T_{ijk}$, is defined as the ratio of the responses between i and j in Equation (3) for an MDOF system. By dividing the two responses, the force $F_{k}\left(\omega \right)$ cancels out. The transmissibility, in this case, is, therefore, a ratio of the frequency response functions (FRF) or transfer functions, H, of the material at i and j with respect only to the location of the force $F_{k}\left(\omega \right)$.

**Figure 3:**
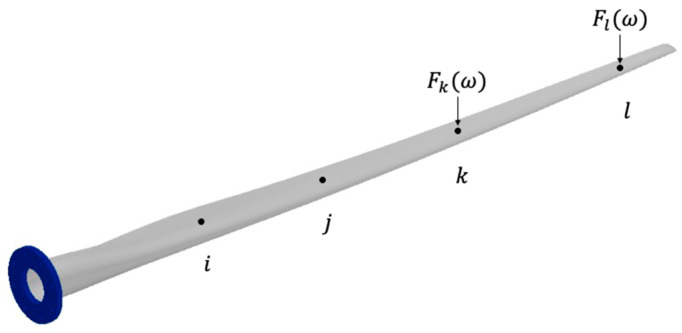
Wind turbine blade with separate forces applied at locations k and l.

This method is useful in cases where the force magnitude is constantly changing, but the location is a known constant. However, the above method only applies to singular forces, so it is limited in its potential applications. Problems arise with this method when the location is unknown, changing, or multiple forces are used. Consider the wind turbine blade in Figure 3, which has two forces applied at two different locations.

(4) $$X_{i}\left(\omega \right)=H_{ik}\left(\omega \right)F_{k}\left(\omega \right)+H_{il}\left(\omega \right)F_{l}\left(\omega \right)$$

(5) $$X_{j}\left(\omega \right)=H_{jk}\left(\omega \right)F_{k}\left(\omega \right)+H_{jl}\left(\omega \right)F_{l}\left(\omega \right)$$

(6) $$T_{ijkl}\left(\omega \right)=\frac{X_{i}\left(\omega \right)}{X_{j}\left(\omega \right)}=\frac{H_{ik}\left(\omega \right)F_{k}\left(\omega \right)+H_{il}\left(\omega \right)F_{l}\left(\omega \right)}{H_{jk}\left(\omega \right)F_{k}\left(\omega \right)+H_{jl}\left(\omega \right)F_{l}\left(\omega \right)}$$

The transmissibility between i and j in this scenario is much more complex as force cancellation does not occur in Equation (6). Information is required on the applied force magnitude and location for transmissibility to be used for damage detection in this scenario.

In [44], the transmissibility was investigated under different force application locations. This research found that when the transmissibility between two sensor outputs was plotted in the frequency domain under the same undamaged condition but with different forces, the transmissibilities converged to the same value at the natural frequencies of the system. This was found to be true using both singular and multiple forces. By restricting the transmissibility analysis to the natural frequencies as in Equation (7) below, the transmissibility becomes completely independent of the magnitude of the applied forces. In addition, the FRFs also become independent of the applied force locations k and l. A system with multiple unknown forces can now be assessed using only the output responses, making this ideal for online damage detection.

(7) $$T_{ij}\left(\omega_{n}\right)=\frac{X_{i}\left(\omega_{n}\right)}{X_{j}\left(\omega_{n}\right)}=\frac{H_{i}\left(\omega_{n}\right)\left(F_{k}\left(\omega_{n}\right)+F_{l}\left(\omega_{n}\right)\right)}{H_{j}\left(\omega_{n}\right)\left(F_{k}\left(\omega_{n}\right)+F_{l}\left(\omega_{n}\right)\right)}=\frac{H_{i}\left(\omega_{n}\right)}{H_{j}\left(\omega_{n}\right)}$$

#### 1.2.4. Damage Detection Using Transmissibility

Transmissibility is dependent on the local mass, stiffness, and damping of the material, making it ideal for damage detection. Changes to the structure due to the presence of flaws would result in a localized change in the transmissibility. Damage detection using transmissibility methods is of interest due to its potential to assess both linear and non-linear types of defects [45]. There are many methods that can be used to assess the changes in transmissibility resulting from a defect. Some examples include the Damage Indicator method [46], Transmissibility Damage Indicator (TDI) [39], and the Damage Index method [45].

Many damage detection methods utilize controlled forces and a large range of frequencies in their research. As such, research often follows the theory described in Equations (1)–(3). As a follow-up to their work in [44], [47] examined logarithmic transmissibility changes using small frequency bands around the natural frequencies. A defect on a cantilever beam was identified using this method at different vibration modes. However, it was noted that some modes were more amenable to identification of the defect than others. The overall importance of frequency band selection was also noted in this work. During numerical testing, different forces applied to the healthy beam revealed that false positive indications of a defect appeared in wider frequency ranges that were not narrowly focused on the natural frequencies. These false positives masked the defect location once the defect was implanted in the structure.

Different types of transmissibility can also be used to assess damage. Much of the literature discussed in this paper focuses on acceleration transmissibility, although other types, such as velocity [48] and strain [49] transmissibility, have also been used. The researchers in [49] argued that strain transmissibility is more sensitive than the other types due to the strain’s relationship with curvature.

Other researchers have focused on the relative merits of various types of signal analysis with transmissibility. Signal amplitudes are predominantly used in the literature. The phase angle was used successfully, along with the signal amplitude in [46], to identify a 5% stiffness reduction added to different elements of a cantilever beam simulation. Research has also been conducted to remove the requirement of transmissibility analysis being conducted in the frequency domain. In [50], strain transmissibility was used to determine damage in the time domain on a simply supported beam set-up. Wavelet transforms have also been used in transmissibility analysis for damage detection in [51].

#### 1.2.5. Transmissibility in Wind Turbine Blades

The transmissibility concept has been applied to damage detection or the characterization of wind turbine blades in a number of different ways. Early research into the topic in [48] assessed velocity transmissibility on a blade using a scanning laser Doppler vibrometer over a large frequency bandwidth. This research was able to recognize an added mass simulating damage but noted a lot of random noise present in the frequency response functions.

A full-size blade undergoing static loading and fatigue testing at the first natural frequency was examined in [52]. An adhesive joint debond between the spar and the shell was successfully identified with accelerometers and FBGs using transmissibility changes of adjacent sensors. This method was also able to monitor the defect’s propagation as it increased in severity. In this experiment, only one defect was present, located in the middle of the blade.

Wavelet energy transforms were used for damage detection on wind turbine blades in [53]. This method was able to determine that two of the three blades tested were damaged, but it did not specify the damage location. These blades were also used in [54] to compare four different system identification models using transmissibility with a similar outcome.

## 2. Materials and Methods

### 2.1. Experimental Set-Up

#### 2.1.1. Blade Design and Fabrication

A wind turbine blade was designed based on the NREL 15MW reference turbine [55]. This blade was scaled from its original length of 117 m to 117 cm (1:100 scale) to maintain practicality for experimental laboratory testing. Additional changes to the scaled blade design included increasing the chord and wall thicknesses to assist with fabrication. A baseplate was also designed to attach the blade to an optical table for vibration testing.

**Figure 4:**
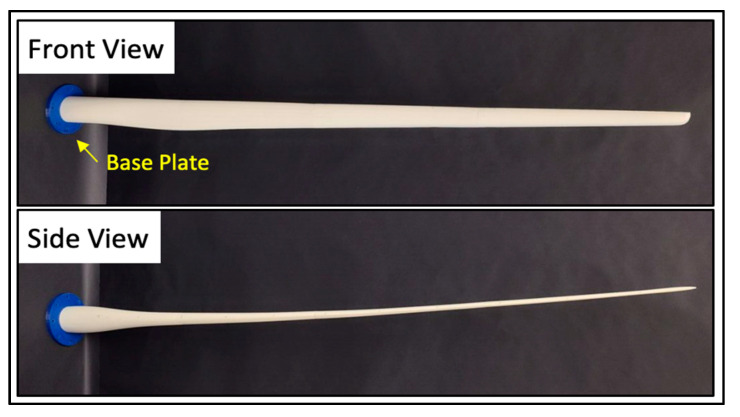
Front and side view of fully assembled wind turbine blade and baseplate.

The blade was 3D printed using the fused filament fabrication (FFF) method. This method allowed for convenient and repeatable fabrication and testing. FFF thermoplastic parts are also easily repairable so that one blade can be repaired and reused many times during experimental testing. The blade was fabricated with a Raise3D Pro2 printer [56] and Raise3D Premium PLA filament [57] (Raise3D, Lake Forest, CA, USA). It was printed in the longitudinal direction to best simulate uniaxial glass fibre. A 0.1 mm layer height and 100% infill were used to best represent the solid material used in the numerical model. The fully fabricated blade assembly is shown in Figure 4.

#### 2.1.2. Blade Joint Adhesion

Due to limitations with print build size, the blade was fabricated in five separate pieces, which were then attached together. Extensive coupon testing was completed to ensure that a blade composed of multiple attached components performed similarly to a blade fabricated in one piece. As the blade was to be subjected only to elastic vibration testing, Young’s modulus (and density) were the key material properties required for the development of an ANSYS numerical model. All other parameters, such as temperature, were assumed to be constant. Full-size 3D printed coupons were compared with half-sized coupons attached together with adhesive; the objective was to confirm the rule of mixtures and that a blade printed as a single unit would show the same vibration characteristics as a blade composed of several segments attached together.

Preliminary testing found that several factors affected the characteristic Young’s modulus of the lab specimens consisting of two half-coupons joined together. The alignment of the half coupons was important in achieving a consistent cross-sectional area and symmetrical stress distribution. The surface roughness, cleanliness, and contact force at the specimen joint were crucial in achieving a sufficient bond. Print location and platform support type were important in maintaining print quality and minimizing variations among coupons. Therefore, all coupons were printed in the same location on the printer with the same ”raft” platform support to minimize differences between prints. A consistent optimal adhesion procedure was also developed using cyanoacrylate glue [58]. The contact surface was first roughened with sandpaper and cleaned with alcohol to remove contaminants. The contact surfaces were assembled and aligned with elastic bands on a backing plate. The adhesive was applied and cured for 24-h under constant force from the elastics. After the elastics and backing plate were removed, a second layer of adhesive was applied to ensure the contact surfaces were fully covered. Once cured, the adhesive cap was sanded until flush with the base material.

**Figure 5:**
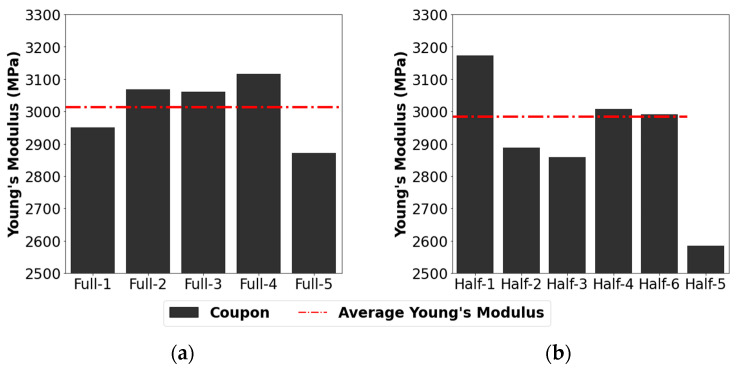
Young’s moduli of (**a**) uniform full-size 3D printed coupons and (**b**) half-sized coupons glued together.

Tensile testing was performed on 5 single-print coupons and 6 glued coupon sets. Figure 5 presents the resultant specimen-averaged Young’s modulus for each coupon. Young’s modulus had averages of 2984 MPa for glued coupons and 3013 MPa for full-sized coupons. The glued coupons had a larger variation in Young’s modulus results, with a standard deviation of 197 MPa compared to a standard deviation of 100 MPa for the full-sized coupons. This was due to imperfections implanted in the blade during the gluing process. Overall, these data show that the adhesion procedure results in no significant change to Young’s modulus compared to a single-print specimen, with a slightly larger variation with the glued samples.

#### 2.1.3. Sensors

**Figure 6:**
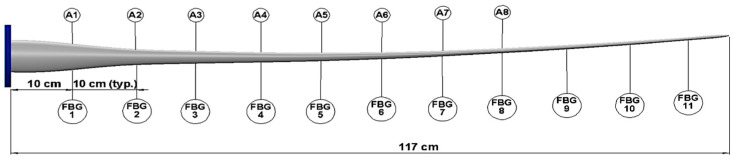
The layout of MPU-6050 accelerometers (A) and fiber Bragg gratings (FBGs) placed along the length of a wind turbine blade. The sensor number corresponds to its location on the blade in 10 cm intervals.

**Figure 7:**
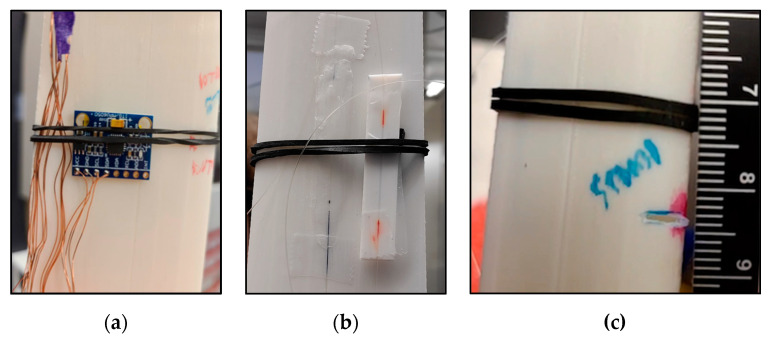
(**a**) Typical MPU-6050 assembly on the blade. (**b**) Typical FBG assembly on the blade. (**c**) Typical 0.5 cm “crack” defect added to the blade. This defect was added 100 cm away from the blade hub.

Two different types of sensors were used in the experimental set-up: eight MPU-6050 micro electro-mechanical system (MEMS) accelerometers [59], positioned 10 cm to 80 cm away from the hub at 10 cm intervals; and eleven fibre Bragg grating (FBG) strain sensors, positioned 10 cm to 110 cm away from the hub at 10 cm intervals. An additional FBG was attached to a 3D-printed coupon separate from the blade for temperature compensation. The accelerometers were attached to the midpoint of the blade’s chord with elastic bands and 3 M tape. The FBGs were spliced together into a single fibre, which was placed in a groove at the midpoint of the blade. The splices were protected with a 3D printed splice cover and attached to the blade with 3 M tape next to the FBGs. Each FBG was adhered to the blade using paraffin wax and tape. Figure 6 and Figure 7a,b show the sensor layout and typical sensor assemblies, respectively. This set-up allowed an assessment of acceleration and strain transmissibility as well as a comparison of the effectiveness of MEMS accelerometers with FBGs.

#### 2.1.4. Defect Inclusion and Repair Procedure

Defects were induced, one at a time, at 10 cm intervals along the blade length from 10 cm to 110 cm away from the blade hub. The defects were in the form of 0.5 cm or 1.0 cm long simulated cracks created using a FEIN Multimaster saw [60]. All “cracks” were approximately 0.1 cm wide and were cut transverse to the blade on the leading edge of the blade’s inside curve. Cracks were chosen for this experiment given their prevalence in the wind industry as either an initial damage to the blade or the result of a different defect type that is allowed to propagate over time. Cracks are also easier to define and quantify than other defect types and are able to be simulated both numerically and experimentally. Figure 7c shows a typical defect.

After the blade’s vibration characteristics corresponding to each defect were measured, the defect was repaired using a MYNT3D printing pen [61] (MYNT3D, Salt Lake City, UT, USA) before the next defect was induced. The repair procedure involved roughening the defect area with sand paper. The region was then cleaned with alcohol to remove contaminants. Raise3D premium PLA filament was applied with the 3D printing pen at 230 °C to promote bonding with the base material. The resulting repair was then ground flush with the blade.

#### 2.1.5. Experimental Testing Procedure

The blade was attached to an optical table using the 3D-printed baseplate and 4 screws. For each blade condition, the blade was struck with an impact hammer at a random location to provide excitation. The vibration response was measured by both sensor arrays using an Arduino Uno R3 [62] (Arduino, Monza, Italy) for the accelerometers and a combination of a laser and interrogator from Ibsen Photonics [63,64] (Ibsen Photonics, Farum, DK) for the FBGs. Separate impact tests were conducted for the accelerometers and the FBGs for each blade condition. The data for each test were filtered and converted to the frequency domain using a fast Fourier transform (FFT) in a Python 3.10.4 script. Five tests were completed for a baseline condition (undamaged blade) as well as for each defect condition. The first three natural frequencies were determined for each test. The natural frequencies selected for transmissibility analysis were based on the median value of the five trials. The transmissibilities of the five trials for each possible pair of sensors were averaged at each median natural frequency for the real, imaginary, amplitude and phase. The transmissibility change between the baseline and each defect condition was then determined for each sensor pair.

### 2.2. ANSYS Numerical Model

#### 2.2.1. Model Set-Up

**Figure 8:**
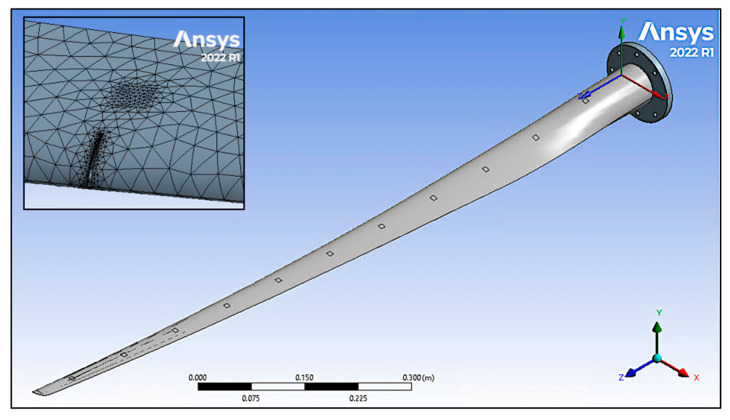
Wind turbine blade in ANSYS 2022 R1 with acceleration and strain sensor locations. A typical meshed 1.0 cm defect is shown in the top left corner. This defect is in line with a simulated sensor. The orientation axis is also shown on the bottom right of the figure.

The CAD model used for 3D printing the blade and baseplate was added to ANSYS Workbench 2022 R1 [65] as a single blade structure. The sensors were modelled as distributed mass components on both sides of the blade. An overview of the simulated blade is shown in Figure 8.

#### 2.2.2. Material

A custom isotropic linear elastic material was created in ANSYS for Raise3D Premium PLA. The Young’s modulus and density were determined using averages obtained experimentally from the 3D-printed adhered coupons discussed in Section 2.1.2. A Poisson’s ratio of 0.33 was used based on research in [66]. A summary of these material values is shown in Table 1.

#### 2.2.3. Defect Inclusion

Transverse through-wall slots of 0.5 cm, which were 1.0 cm in length and 0.1 cm in width, were added to the model. Each defect was added in 10 cm intervals (in line with the sensors) at the leading edge on the upper side of the blade. Figure 8 shows a typical 1.0 cm defect.

#### 2.2.4. Mesh

Tetrahedron elements that were 5 mm, 1 mm, and 10 mm were used for the body, sensor locations, and baseplate, respectively. Defects were primarily modelled using 1 mm mesh elements, but some were subsequently adjusted to achieve convergence. A typical blade mesh is shown in Figure 8.

To test whether these mesh sizes were appropriate, the modal analysis results for the undamaged ANSYS model were compared with the average experimental results of the pristine baseline blade. This comparison is shown in Table 2. All three simulated natural frequencies were within 1% of the experimental results, and the mesh geometry was, therefore, deemed adequate.

#### 2.2.5. Numerical Simulations Using Modal and Harmonic Analyses

The numerical transmissibility simulations were performed using two features of the ANSYS workbench. First, modal analysis was used to determine the natural frequencies. The harmonic response function was then used to apply an input force of random magnitude and location to the simulated blade. The output response was calculated at frequencies from 0 to 50 Hz in increments of 0.05 Hz to capture the first three natural frequencies. A damping ratio of 1.2% was used as suggested by [67]. Transmissibility values were then calculated for each possible sensor pair using the real, imaginary, amplitude and phase responses at each natural frequency.

### 2.3. Total Method

Transmissibility research such as [47,49] primarily assessed transmissibility changes using pairs of sensors that were adjacent to each other. Comparing adjacent pairs was found to be effective with varying damage identification methods.

When a flaw is implemented, transmissibility changes also occur with other pairs of sensors within the sensor matrix. For example, if a defect occurs near sensor i, a change in transmissibility will be found in all sensor pairs associated with sensor i, not just between sensor i and its adjacent sensors. The Total Method in Equation (9) uses a variation of the Damage Indicator from [46] in Equation (8) and sums all the transmissibility changes from all the available pairs for each sensor at each natural frequency. This formula yields a value, $Y_{i},$ for the total transmissibility change for a sensor in the sensor matrix, where m is the total number of sensors that can be paired with sensor i.

(8) $$D_{ij}(\omega_{n})=\frac{T_{ij_{Damaged}}\left(\omega_{n}\right)-T_{ij_{Undamaged}}\left(\omega_{n}\right)}{T_{ij_{Undamaged}}\left(\omega_{n}\right)}$$

(9) $$Y_{i}\left(\omega_{n}\right)=\sum_{j=1}^{m}D_{ij}\left(\omega_{n}\right)$$

With the Total Method, the transmissibility changes are amplified as data from multiple sensors are used. This results in a higher total transmissibility change than when using just the adjacent sensors, which makes it easier to identify a defect. The Total method was chosen for this experiment as it provides a higher sensitivity to small defect identification while suppressing any noise effects emanating from a single sensor. This was crucial for the defect sizes chosen for the evaluation of the transmissibility concept on the wind turbine blade.

## 3. Results

### 3.1. Strain Transmissibility

#### 3.1.1. Variables

Non-destructive defect detection on the model turbine blade was assessed by means of strain transmissibility using combinations of variables from four different categories: defect size, vibration mode, strain orientation, and signal type. A total of 24 different variable combinations were used to analyze the transmissibility changes experimentally along a single axis with the FBGs. The ANSYS model was able to examine the strain in all three axes, so 72 variables were tested numerically. These variable combinations are listed in Table 3 and were all assessed to determine which best determined the existence and location of a flaw. Further analysis of the viability of the strain transmissibility concept was then pursued for this optimal choice of the four variables.

#### 3.1.2. Defect Identification Threshold and Confusion Matrix Development

**Figure 9:**
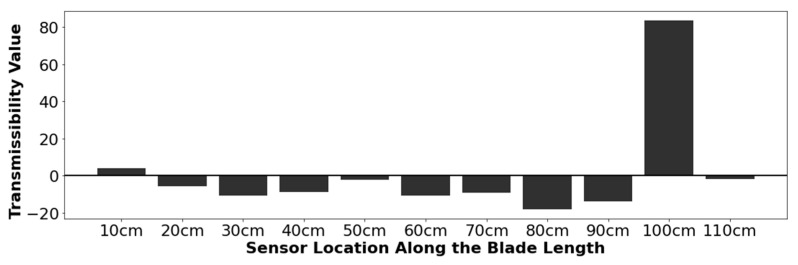
Experimental (Z direction) mode 1 amplitude results for a 1.0 cm transverse defect. The defect is located at 100 cm.

Qualitative analysis of the strain transmissibilities found that damage could be identified and located in select models by identifying the sensor with the largest total method value. An example of the experimental results is shown in Figure 9. In this figure, a 1.0 cm defect is added to a location 100 cm from the blade hub, and the total method values are calculated at each of the 11 sensors using Equations (8) and (9). The maximum value for this dataset is noted to occur at 100 cm—the location of the defect. 

A damage threshold value is required to properly analyze the blade condition with different defect sizes in different locations. This value can be used to simplify the damage detection algorithm to a binary response function. If the total method value at a sensor is above the threshold, damage is assumed to be present at this location and a value of one is returned. If the total method value at a sensor is below the threshold, damage is assumed to be absent at that location and a value of zero is returned. An ideal threshold value should be small enough to identify defects in as many locations as possible. It must, however, also be a large enough value to minimize false positive identifications of defects at other sensor locations.

A confusion matrix analysis was used to determine an ideal threshold value for each set of variables listed in Table 3 and identify the combination that could most reliably detect damage with minimal occurrence of false positives. The confusion matrix determines the true positives (TP), true negatives (TN), false positives (FP) and false negatives (FN) when comparing a set of data to an ideal case [68]. Eleven defects were assessed for each possible combination of variables. The results from each variable combination were compared to an 11 × 11 matrix of zeros with ones on the diagonal (ones at each damage location in the ideal case and zeros in all other locations).

The “accuracy” and “precision” score metrics from Scikit-Learn [69] were used to identify the best overall model. The accuracy equation looks at the overall predictability. The precision gives an indication of the potential presence of false positives when compared to true positives. These equations are shown below.

(10) $$Accuracy=\frac{TP+TN}{TP+TN+FP+FN}$$

(11) $$Precision=\frac{TP}{TP+FP}$$

#### 3.1.3. Experimental Results

Experimental results for detecting blade damage will be examined first to characterize the effect of each of the four identified variables on the output of the confusion matrix.

##### 0.5 cm Results

**Figure 10:**
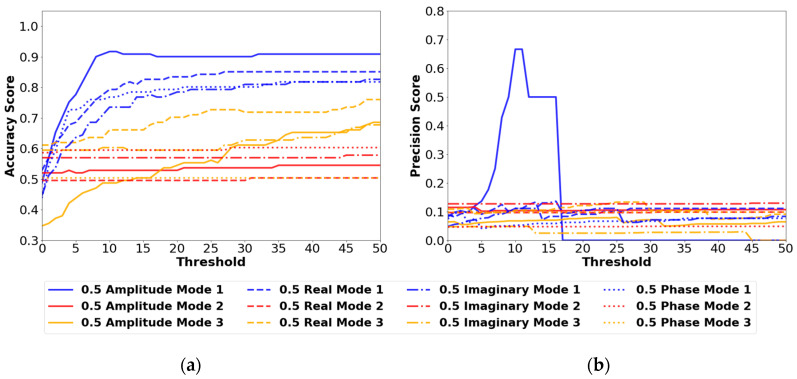
(**a**) Accuracy results and (**b**) precision results using the 0.5 cm defect size and the vibration mode and signal type variables from Table 3. All results are from the FBGs orientated in the Z direction.

Figure 10 summarizes the results of the confusion matrix outputs for each set of parameters in Table 3 for the 0.5 cm defect. The best performing set of variables in this plot is the amplitude mode 1 set, which achieves accuracy scores above 0.9. The challenge with identifying the 0.5 cm defect is shown in the precision plot. In Figure 10b, Amplitude mode 1 also has the highest precision score with a maximum score of 0.67 occurring between threshold values of 10 and 11. This score indicates a high percentage of false positives occurring relative to the number of true positives the model can identify. Furthermore, the precision score decreases as the threshold value increases, which is an indication that the maximum total method values for the 0.5 cm defect are very small. True positives become false negatives as the damage threshold is increased above the maximum total method value. The precision score decreases as it is dependent on the number of true positives, as shown in Equation (11). Although the amplitude mode 1 set of parameters can identify a small range of defects (90–100 cm) at a threshold of 10–11, it is not recommended to use due to the low precision score. Overall, the 0.5 cm defect was determined to be too small to be accurately and precisely identified with any of the strain transmissibility methods.

##### 1.0 cm Results

**Figure 11:**
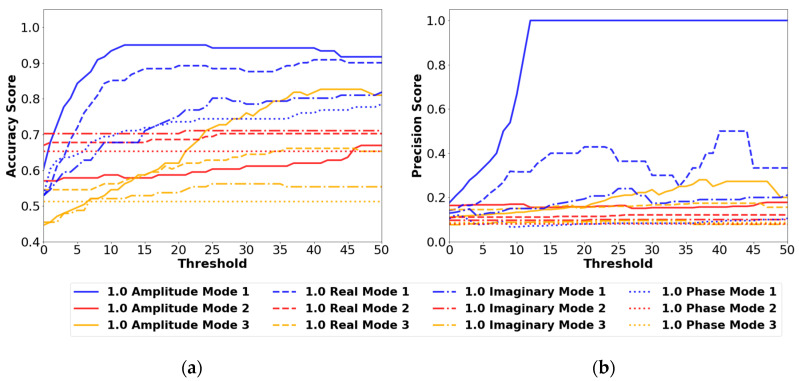
(**a**) Accuracy results and (**b**) precision results using the 1.0 cm defect size and the vibration mode and signal type variables from Table 3. All results are from the FBGs orientated in the Z direction.

The larger-sized defect proved easier to identify, with higher accuracy and precision scores compared to the 0.5 cm defect. The 1.0 cm results are shown in Figure 11.

The maximum accuracy score in Figure 11 occurs at a threshold between 12 and 24 for the mode 1 amplitude model. This model performs the best out of all the available options. The precision score for this model increases to a value of 1 after a threshold value of 12, indicating that no false positives are occurring with this model.

**Figure 12:**
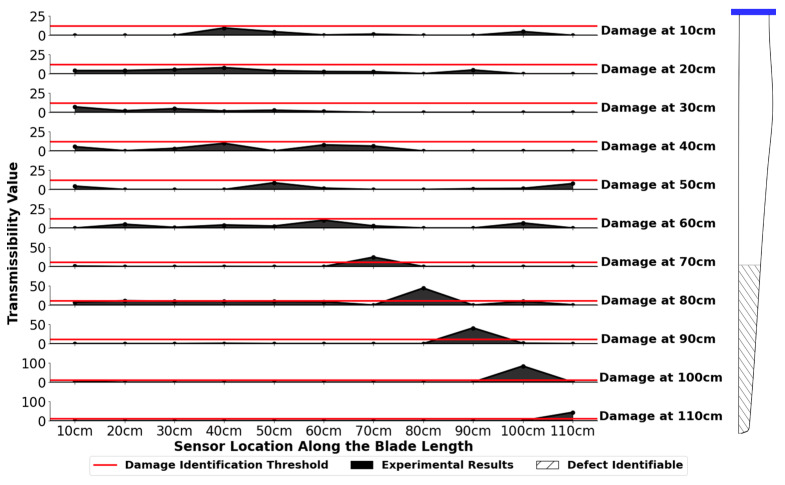
Mode 1 amplitude experimental results for the 1.0 cm defect at 11 locations 10 cm to 110 cm away from the hub. A damage threshold of 12 is added, and the identifiable defect range of 70 cm to 110 cm is shown on the wind turbine blade. Note that the data points are connected in these plots only to aide with visualization, and all negative sensor values are set to zero.

The Total Method transmissibilities for all 11 defect locations for the mode 1 amplitude variable combination is shown in Figure 12 for the 1.0 cm defect. This plot depicts all the positive Total Method results. All negative sensor results were set to zero to aide in plot visualization. In this figure, maximum total method values are noted at a number of sensors corresponding to the damage location. This maximum value increases as the defect moves away from the hub and toward the tip of the blade. Closer to the blade hub, the peak values are less prominent, and the damage location is difficult to identify compared to nearby sensor locations. The implementation of a threshold removes these locations from the resulting defect identification range as the maximum total method value is too small to reliably identify the damage location at this defect size.

Figure 12 shows that defects can be successfully located at a range of 70 cm to 110 cm for the 1.0 cm defect utilizing a threshold value of 12. At any threshold value between 12 and 24, this location range can be identified. This model is both accurate and precise per the confusion matrix analysis and is, therefore, suitable for detecting defects in this range. This is a promising result, as many wind turbine defects occur close to the tip of the blade.

#### 3.1.4. Numerical Results

The numerical model results developed with ANSYS are examined next. First, a comparison is made between the numerical model and the highest performing experimental model to assess the feasibility of the ANSYS model for defect detection. The numerical results of the other variable combinations are also reviewed.

##### Comparison with Experimental Results

**Figure 13:**
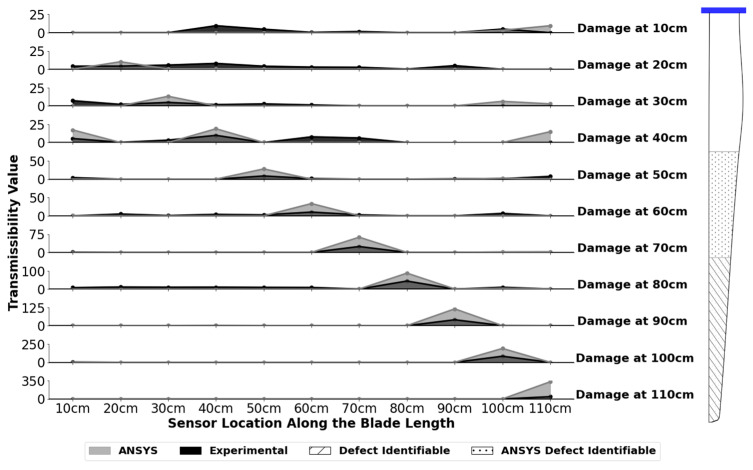
Comparison of the Z direction ANSYS numerical model results with the experimental results for the 1.0 cm defect and the mode 1 amplitude parameters. Defects are located at 11 locations 10 cm to 110 cm away from the hub. The defect ranges of 40–110 cm numerically and 70–110 cm experimentally are shown on the wind turbine blade. Note that the data points are connected in these plots only to aide with visualization and all negative sensor values are set to zero.

Given that experimentally the best result was based on the mode 1 amplitude variable combination with sensors oriented in the Z direction, the ANSYS numerical model was first assessed under these conditions to determine if it achieved similar results. The numerical results showed that like the experimental model, a peak value occurred at the sensor closest to the damage. Significantly larger total method values occurred in the numerical model compared to the experimental results. This is shown in Figure 13 for the 1.0 cm defect size.

**Figure 14:**
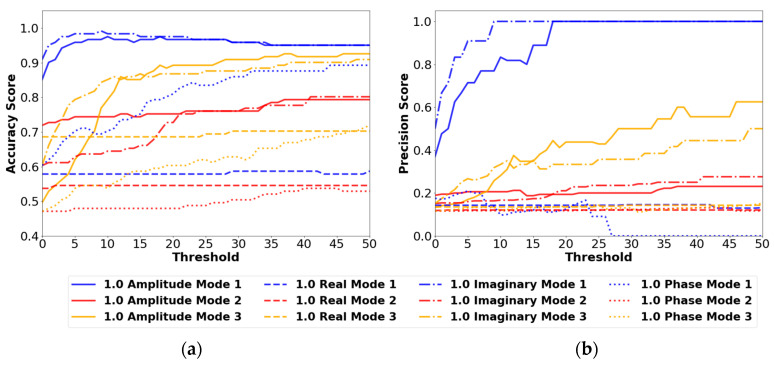
(**a**) Accuracy results and (**b**) precision results using the 1.0 cm defect size and the vibration mode and signal type variables from Table 3. All results are from the ANSYS numerical model with simulated sensors orientated in the Z direction.

A confusion matrix analysis found that the numerical model required a higher threshold to eliminate the false positives versus the experimental model. This was likely due to the larger total method values produced overall by the numerical model at all possible sensor locations. A threshold value of 18 obtained the highest accuracy score of 0.975 and the highest precision score of 1.0. This is shown in Figure 14. The larger total method values in the numerical model also yielded an increased defect range. A 1.0 cm defect is identifiable in ANSYS at locations ranging from 40 cm to 110 cm from the blade hub; this range encompasses the successful experimental result range of 70 cm to 110 cm. The ANSYS numerical model is therefore a useful tool for planning experimental NDT studies to identify defects with a minimum size of 1.0 cm. Caution should be taken when assessing defects located in the 40 cm to 60 cm range numerically, as the expected experimental values will be much smaller and may not exceed the damage threshold for identification.

##### Other Variables

The amplitude mode 1 model performed the best overall experimentally and was also a high performing model numerically. However, the highest performing model numerically was the imaginary mode 1 variable combination as shown in Figure 14 for the 1.0 cm defect. The corresponding experimental results were poor for this set of parameters. Further improvements to the experimental methods and signal analysis may provide better results when using the imaginary portions of the signal in future research.

The numerical model was able to obtain results for the 0.5 cm defect at some locations, in part due to the overall higher total method values outputted by the numerical model. Like the experimental model, the threshold value with the highest accuracy and precision was only useful at identifying a few damage locations. Although more accurate and precise than the experimental model, the numerical model is also not recommended for predicting defects at a size of 0.5 cm or less. The reliable damage location range is not large enough for this model to be effective.

The X and Y strain orientations were also examined with the numerical model. In the confusion matrix analysis of the 1.0 cm defect, both directions performed similarly to the Z direction, with both the amplitude and imaginary responses in the first mode. In a direct comparison of the outputs of these models, the X-orientation model produced slightly higher total method values at the damaged sensors, indicating that this orientation is potentially more sensitive to this type of defect. Further experimental testing is recommended to assess the strain transmissibility results in the X direction.

### 3.2. Acceleration Transmissibility

#### 3.2.1. Variables

Flaw detection via acceleration transmissibility was assessed using the same variable categories as those for strain transmissibility: defect size, vibration mode, acceleration orientation, and signal type. Table 4 presents the variables examined. A total of 72 different variable combinations were used in ANSYS numerical analysis. However, initial experimental results with MPU-6050 accelerometers showed that the acceleration in the Z direction had the least excitation; it was therefore eliminated from further data collection and analysis to reduce the sampling time. As a consequence of that decision, only 48 variable combinations were assessed experimentally for acceleration in the X and Y orientations. In addition, defect locations for the experimental tests ranged only from 10 cm to 80 cm away from the blade hub, as the maximum number of MEMS accelerometers was limited to eight with the available experimental testing equipment.

#### 3.2.2. Experimental Results

Experimentally, neither of the 0.5 cm and 1.0 cm defect sizes were detectable at any location on the model blade using the variable combinations described in Table 4. The data acquired from the MPU-6050 sensors were random, and no patterns were noted using qualitative methods. The assessment of the ideal variable combination was carried out using ANSYS simulations, and the differences between the experimental and numerical results for acceleration are discussed in Section 4.3.

#### 3.2.3. Numerical Results

**Figure 15:**
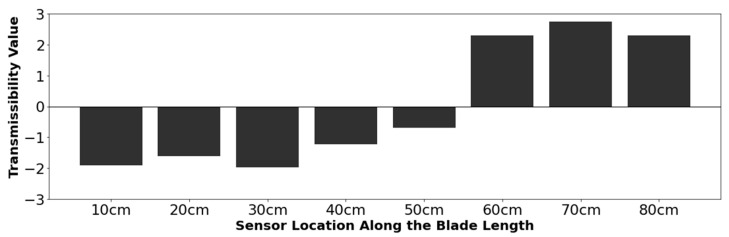
Numerical mode 1 results (imaginary signal component) for a 1.0 cm transverse defect with sensor orientation in the Y direction. The defect is located at 50 cm from the blade hub.

The numerical results were examined qualitatively to determine if a pattern existed for locating defect damage using acceleration transmissibility and the total method as described in Section 2.3. Several different patterns emerged from the ANSYS numerical transmissibility results that could be used to indicate the presence and location of a defect. An example is shown in Figure 15, where the total method value for each sensor was calculated using Equations (8) and (9). 

For the results shown in Figure 15, the transmissibility readings are positive for all the sensors located at a distance greater than 50 cm—the location of the damage. Negative transmissibility values are present for all sensors from 10 cm to 50 cm. For the acceleration data, the defect’s location can be identified by determining where the data intercept the X-axis. Most variable combinations with tangible results followed the pattern in Figure 15. However, some combinations showed an inverted pattern, where the sensors before the damaged sensor were positive, and negative values occurred after the sensor closest to the damage location. Both of these pattern types were assessed with a confusion matrix for all the variable combinations shown in Table 4. The results presented below are for the pattern shown in Figure 15, as this pattern was more prevalent and had the highest accuracy scores overall.

**Figure 16:**
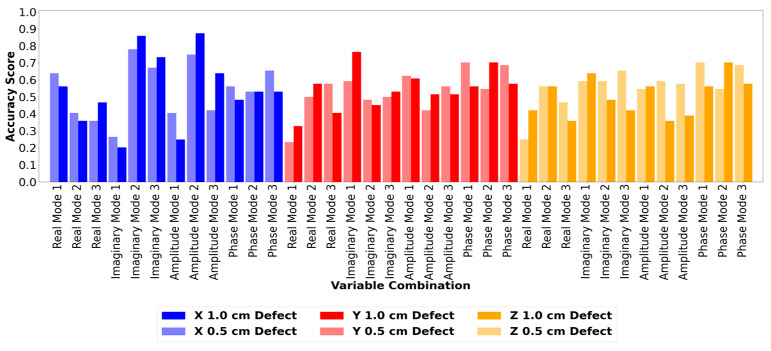
Accuracy score comparison of all 72 variable combinations for the ANSYS numerical model.

Based on the qualitative analysis, an initial damage threshold of zero was used to determine the damage location. All total method values that were below zero were set to zero, and all total method values above zero were set to one and compared to an ideal results matrix. In an ideal 8 × 8 matrix assuming perfect results, all sensors before and including the damaged sensor yield a value of zero (below the threshold). All sensor locations after the defect yield a value of one (above the threshold). The accuracy scores of the resulting confusion matrices were calculated using Scikit-Learn and Equation (10). The accuracy score was used as the deciding factor for the best overall model as the acceleration results rely on the outputs of multiple sensors to identify each damage location instead of a singular sensor output. The more sensors that match the ideal matrix outputs overall, the easier it will be to identify the damage location. The accuracy scores of all 72 variable combinations are shown in Figure 16.

**Figure 17:**
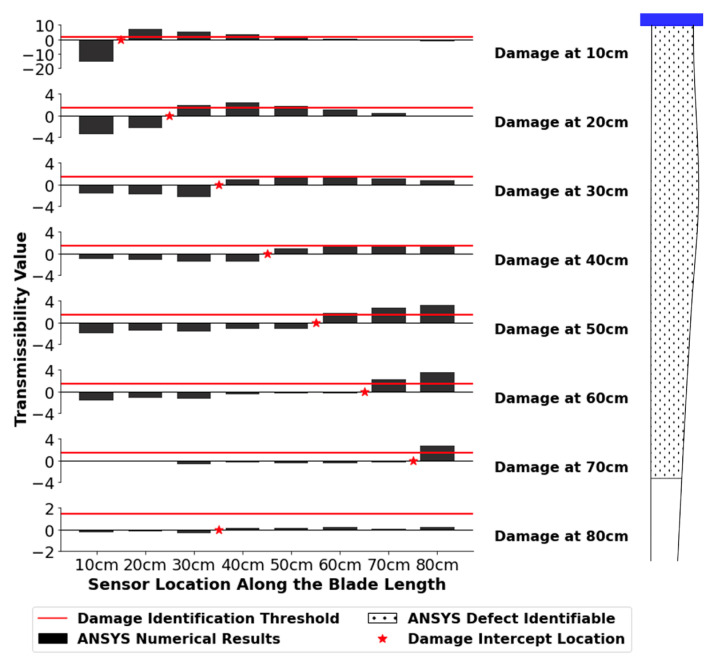
X amplitude mode 2 results for the 1.0 cm defect for acceleration transmissibility. The horizontal line is the identification threshold where damage has occurred and is set to 1.4. The intercept locations are marked with red stars to identify damage locations. The identifiable defect range of 10 cm to 70 cm is shown on the wind turbine blade.

Figure 16 shows that only a few select variable combinations have accuracy scores in excess of 0.7. This indicates that very few combinations can clearly identify the defect location at any given defect size. The majority of the variable combinations assessed produced random results. The best performing set of parameters was X amplitude mode 2, with a defect size of 1.0 cm. This variable combination had the highest accuracy score of 0.875, indicating that it would be the most reliable at identifying the location of a flaw numerically. The equivalent variable combination had an accuracy score of 0.75 with a 0.5 cm defect. This indicates that a relationship exists between defect size and accuracy for this set of parameters, where the larger the defect, the higher the accuracy score. The detailed results for the X amplitude mode 2 parameter combination are shown in Figure 17 for the 1.0 cm flaw size. Further analysis of the results for the detection of 0.5 cm flaws was not pursued due to the relatively poor success rate for locating defects of this size.

In Figure 17, the total method values change from negative to positive at the sensor closest to the damage when a 1.0 cm defect is added. Each intercept location is marked with a red star. The location of the defect can, therefore, be determined. A defect location range of 10–70 cm can be correctly identified with the X amplitude mode 2 results.

Although the location of the 1.0 cm defect can be determined through numerical modelling, an additional threshold was required to minimize the probabilities of “false positives” so that this model could be of practical use. The maximum Total Method values were examined for the X amplitude mode 2 parameter set, and a value of 1.4 was selected as the threshold to indicate if a defect of 1.0 cm or greater had occurred. This was the highest possible threshold that would capture the presence of a defect over the full location range of 10 cm to 70 cm. Given that the overall transmissibility changes due to a defect are very small and, therefore, more susceptible to noise, the threshold needs to be as high as possible to minimize the likelihood of false alarms.

Based on numerical modelling, overall cumulative transmissibility values are much smaller using acceleration versus strain. This makes the presence of a defect more difficult to assess with the acceleration model. The acceleration algorithm is also much more complex, with two thresholds required to identify that the blade has a defect, as well as determine its location. The primary advantage of utilizing acceleration transmissibility versus strain transmissibility is in *locating* the defect.

The strain transmissibility results were more sensitive to defect locations closer to the tip of the blade. As shown in Figure 17, the numerical acceleration transmissibility can be used to locate a defect situated in the range of 10–70 cm, with more prominent transmissibility changes occurring when the defect location is closer to the blade hub. The acceleration transmissibility results would be useful for defects in this range as they are not covered by the range of strain transmissibility. Acceleration transmissibility, in tandem with strain transmissibility, can increase the overall defect identification range on the blade. However, further research is required experimentally to properly validate the numerical acceleration results.

## 4. Discussion

### 4.1. Transmissibility Analysis vs. Natural Frequency Analysis

NDT, through vibration analysis, often relies on monitoring changes to the natural frequencies of the structure caused by damage. This method is not sensitive enough to detect the 1.0 cm blade damage considered in this study. For all defect sizes and locations tested, a maximum change of only 0.58% was noted at the first three natural frequencies from the experiments. The ANSYS simulations also indicated a very small change in the natural frequencies of 0.32% across the first three natural frequencies. These frequency changes are too minute to detect small defects. Much larger defects could significantly shift the natural frequencies and signal potential issues, albeit at a stage that would necessitate more extensive repairs.

The signal amplitudes can also be used in vibration monitoring to ensure that blade damage does not exceed a level that could compromise the blade’s structural integrity. However, for a system where the applied force magnitude and location are entirely random, it is difficult to appropriately compare the amplitudes of two different signal outputs to identify damage.

This study demonstrated that the transmissibility method may be able to locate a 1.0 cm defect at certain locations along the length of the blade and can be considered a worthwhile alternative to traditional vibration monitoring methods. The downside to transmissibility is the number of sensors required to perform this analysis. Regular vibration monitoring can be completed by one or two sensors placed in each direction to assess amplitude or natural frequency changes. Transmissibility analysis requires an array of sensors across the structure to properly identify and locate the defect.

### 4.2. Transmissibility Analysis at the Natural Frequencies

As discussed in Section 1.2.3, the transmissibilities at the natural frequencies can be used for defect detection regardless of the applied blade excitation force characteristics. The theory suggests that all vibration modes offer a similar potential to characterize a flaw and that it is possible to detect any size of a defect. In practice, certain vibration modes proved more effective than others in detecting defects, and a vibration sensor’s sensitivity and orientation also influenced its flaw detection ability. The results from this project found that a minimum defect size was required in order for the changes in transmissibility at the natural frequencies to be detected by the vibration sensors.

The ANSYS numerical simulation found that the first and third vibrational modes of the blade are primarily associated with displacements oriented in the flap-wise Y direction. The second mode primarily featured displacements along the edgewise X direction. When performing NDT through transmissibility analysis, it must be considered that the application of various random forces will not equally excite the blade in all directions.

Repeatable and accurate measurements of natural frequencies associated with the second and third vibrational modes were very difficult in a laboratory setting, likely due to a lack of strong excitation of these modes by the applied impact force. In practice, field testing with the given wind turbine geometry and weather patterns can determine which vibrational modes are adequately excited for acceleration and strain transmissibility analysis. In our set-up, mode 1 was clearly the best candidate as it was the only mode to yield useful damage information experimentally while using strain transmissibility.

**Figure 18:**
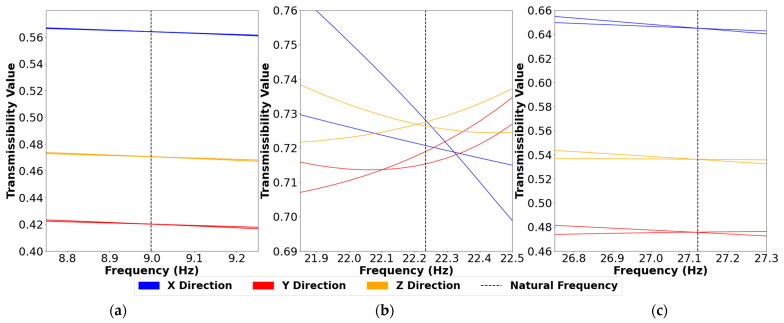
ANSYS strain amplitude transmissibilities calculated between the 10 cm and 20 cm sensors with two different random forces applied and plotted with respect to frequency. Each plot contains the X, Y and Z directional transmissibilities at the (**a**) first, (**b**) second and (**c**) third natural frequencies. Each natural frequency is shown as a dotted vertical line.

Figure 18 shows the ANSYS model strain amplitude transmissibilities in the X, Y, and Z directions between sensors at 10 cm and 20 cm from the blade hub of an undamaged blade subjected to two different random forces.

In the first mode, the strain transmissibilities remain relatively constant around the natural frequency in all three directions, regardless of the magnitude and location of the applied forces. Transmissibility variations are slightly larger in the third vibration mode but still converge to the natural frequency in all orientations. This suggests that when using modes one and three, correct identification of the natural frequency is not critical. The X and Y strain transmissibility pairs do not converge at the second natural frequency, which demonstrates the importance of the sensor’s measurement direction. The X and Y directions fail to identify defects in the second mode as they do not follow the theory in Equation (7), and the transmissibility values depend on the applied force. The Z direction transmissibilities converge close to the second natural frequency but diverge at frequencies that are immediately adjacent. Therefore, to use the mode two transmissibility in the Z direction, one must accurately identify the natural frequency, as inherent transmissibility variations around the natural frequency can obscure those caused by damage. Experimentally, accurate identification of the second mode natural frequency was difficult; this explains the inability to detect defects using strain transmissibility with the second vibration mode.

### 4.3. Strain Transmissibility vs. Acceleration Transmissibility

The strain transmissibility results were easier to interpret than the acceleration results for the transverse defects tested experimentally and modelled numerically. A one-step algorithm could consistently locate the defect under all orientations, modes and signal types by identifying the sensor with the highest total method value. In contrast, the acceleration-based models required a two-step process: first to identify the presence of a defect and then to locate it. Different damage identification patterns emerged in the qualitative analysis of the acceleration numerical model depending on the selected variables for analysis. This added an additional challenge in creating an algorithm for the acceleration transmissibility analysis that would be suitable for all variables.

Experimentally, only the strain transmissibility was effective at locating defects. The acceleration transmissibility was unable to identify damage at any of the locations tested. The numerical models showed that acceleration transmissibility was viable for damage detection, but the overall total method values achieved were miniscule for both the 0.5 cm and 1.0 cm defects.

As shown in comparison between the numerical and experimental methods for strain transmissibility analysis, the total method values in the numerical model were significantly larger than those obtained through experiments. The same numerical model was used for the acceleration analysis. As strain and acceleration are related through a time derivative and Hooke’s Law, this suggests that the true acceleration results are perhaps much smaller than the numerical prediction. Experimental noise and artifacts may have obscured the defects. This indicates that the minimum detectable defect size is potentially larger than 1.0 cm for acceleration transmissibility analysis or that a more sensitive accelerometer is required. Further research is required, particularly with larger defects and alternative sensor types, to experimentally validate the acceleration transmissibility method.

The fibre optic technology in the case of strain sensing was also advantageous over the MEMS-based acceleration sensors as multiple FBGs were attached in series on a single fibre, and the final set-up was much lighter than its accelerometer counterpart. The sampling rate of the fibre optic interrogator could also support a much higher frequency range. The MPU-6050 accelerometers, while having the advantages of lower cost and being multi-directional, were limited by the maximum achievable sampling rate of the Python and Arduino operating codes. As a result, the number of accelerometers was limited to eight, and only two of three acceleration orientations could be sampled. These changes allowed the equipment to sample at a rate high enough to analyze the first three natural frequencies without being subject to aliasing.

### 4.4. Total Method Effectiveness

The Total method for processing vibration signals was found to be effective for locating transverse defects at sensor locations. It is feasible as a less complex transmissibility calculation than other methods found in research. This method utilized signals from all sensors to amplify the transmissibility changes at a defect location, making it easier to identify defects than using only adjacent sensor pairs. As each output is dependent on all the sensors within the network, this method can be susceptible to erroneous readings from a defective sensor, which can mask the defect.

A direct comparison between the Total Method and other transmissibility calculations was outside of the scope of this project. Further research is recommended comparing the Total method to other signal analysis methods found in the literature to determine the most effective transmissibility calculation technique for damage detection.

### 4.5. Comparison with Other Transmissibility Research

Natural frequency-based transmissibility originally discussed in [44,47] was validated in this research for damage detection. This experiment assessed the transmissibility changes at each exact natural frequency instead of examining changes at a frequency range around each natural frequency. The effects of sensor orientation, signal type, defect size, location and defect size were also examined in this research. The damage imparted to the test structure was also not large enough to shift any of the lower vibrational modes in this case.

It was argued in [49] that strain transmissibility is more sensitive to damage than acceleration transmissibility. The experiments in this paper aligned with this argument for both the numerical and experimental results. Strain transmissibility was more sensitive to the smaller defect size overall, but this could potentially be explained by the difference in sensors used experimentally. Fewer steps were also required to analyze the strain experimental data versus the acceleration data, making the strain transmissibility method more practical for use.

In [52], the focus of the transmissibility experiments was on the propagation of a defect in the middle of the blade resulting from a fatigue test. The research in this paper expanded on this by examining defects in different locations and by looking at multiple natural frequencies. This research also developed a threshold that could be used to assess that damage had occurred, which would enable this transmissibility method to be used for online monitoring.

## 5. Conclusions

This project was able to identify a transverse defect on a 3D-printed wind turbine blade using transmissibility analysis. This research found that the amplitude results were the best at identifying the defect using strain transmissibility at the first natural frequency. This variable combination was able to detect experimentally a 1.0 cm defect at a range of 70 cm to 110 cm away from the blade hub. This range was determined using a damage threshold value, which identifies whether a defect is present. A numerical model was completed and verified with the strain experimental model in the Z direction.

Acceleration transmissibility was unable to identify a defect experimentally. Numerically, the 1.0 cm defect was best identified with the amplitude results in the X direction at the second natural frequency. It was found that the numerical model required a two-step algorithm to both identify that a defect had occurred and determine the location using acceleration transmissibility.

Overall, this research shows that transmissibility is promising as an online method of damage detection for wind turbine blades as a small-sized defect was able to be identified at a natural frequency and thus be independent of the unknown applied forces to the wind turbine blade during operation. The transmissibility analysis used in this project found limitations in the size of the defect it could identify with the scaled blade. The effectiveness of transmissibility when examining other defect parameters is currently unknown. This research also found that the natural frequencies of the blade were not equally successful at identifying the defect. Careful natural frequency selection is required to use the transmissibility method. Future work is currently in progress testing additional crack sizes, orientations and locations on the blade to assess the effectiveness of transmissibility and further identify its advantages and limitations. Additional research is also required to test other defect types at different locations and orientations on the blade to assess their relationship with the natural frequencies prior to conducting tests on in-service blades.

## Figures and Tables

**Table 1:** PLA parameters used in ANSYS.

Young’s Modulus (MPa)	Density (kg/m^3^)	Poisson’s Ratio
2984	1128	0.33 [66]

**Table 2:** Natural Frequencies for the baseline undamaged ANSYS simulation and experiments.

Vibration Mode	ANSYS Natural Frequency (Hz)	Experimental Natural Frequency (Hz)
1	8.998	8.990
2	22.234	22.133
3	27.122	27.150

**Table 3:** Strain Transmissibility Variables.

Variable Type	FBG Experimental Variables Examined	ANSYS Numerical Variables Examined
Defect Size	0.5 cm, 1.0 cm	0.5 cm, 1.0 cm
Vibration Mode Number	1, 2, 3	1, 2, 3
Strain Orientation	Z	X, Y, Z
Signal Type	Real, Imaginary, Amplitude, Phase	Real, Imaginary, Amplitude, Phase

**Table 4:** Acceleration Transmissibility Variables.

Variable Type	MPU-6050 Experimental Variables Examined	ANSYS Numerical Variables Examined
Defect Size	0.5 cm, 1.0 cm	0.5 cm, 1.0 cm
Vibration Mode Number	1, 2, 3	1, 2, 3
Strain Orientation	X, Y	X, Y, Z
Signal Type	Real, Imaginary, Amplitude, Phase	Real, Imaginary, Amplitude, Phase

## Data Availability

The data are available on request.
